# Safety, effectiveness, and cost of dipeptidyl peptidase-4 inhibitors versus intermediate acting insulin for type 2 diabetes: protocol for a systematic review and network meta-analysis

**DOI:** 10.1186/2046-4053-2-47

**Published:** 2013-06-28

**Authors:** Andrea C Tricco, Jesmin Antony, Charlene Soobiah, Brenda Hemmelgarn, David Moher, Brian Hutton, Catherine H Yu, Sumit R Majumdar, Sharon E Straus

**Affiliations:** 1 Li Ka Shing Knowledge Institute, St. Michael’s Hospital, 209 Victoria Street, East Building, Toronto, Ontario M5B 1T8, Canada; 2 Departments of Medicine and Community Health Sciences, University of Calgary, 2500 University Dr NW, Calgary, Alberta T2N 4Z6, Canada; 3 Clinical Epidemiology Program, Centre for Practice-Changing Research, Ottawa Hospital Research Institute, 725 Parkdale Ave, Ottawa, Ontario K1Y 4E9, Canada; 4 Department of Medicine, University of Alberta, 116 St. and 85 Ave, Edmonton, Alberta T6G 2R3, Canada; 5 Department of Geriatric Medicine, University of Toronto, 27 Kings College Circle, Toronto, Ontario M5S 1A1, Canada

**Keywords:** Systematic review, Type 2 diabetes, Dipeptidyl peptidase-4, Intermediate-acting insulin, Glycosolated hemoglobin

## Abstract

**Background:**

Type 2 diabetes mellitus (T2DM) results from insulin resistance and relative insulin deficiency. T2DM treatment is a step-wise approach beginning with lifestyle modifications (for example, diet, exercise), followed by the addition of oral hypoglycemic agents (for example, metformin). Patients who do not respond to first-line therapy are offered second-line therapy (for example, sulfonylureas). Third-line therapy may include insulin and/or dipeptidyl peptidase-4 (DPP-4) inhibitors.

It is unclear whether DPP-4 inhibitors are safer and more effective than intermediate acting insulin for third-line management of T2DM. As such, our objective is to evaluate the comparative effectiveness, safety and cost-effectiveness of DPP-4 inhibitors versus intermediate acting insulin for T2DM patients who have failed both first- and second-line diabetes treatments.

**Design/Methods:**

Electronic searches of MEDLINE, Cochrane Central Register of Controlled Trials, EMBASE, and grey literature (for example, trial registries, public health websites) will be conducted to identify studies examining DPP-4 inhibitors compared with each other, intermediate acting insulin, no treatment, or placebo for adults with T2DM. The outcomes of interest include glycosylated hemoglobin (A1C) (primary outcome), as well as emergency department visits, physician visits, hospital admissions, weight gain, quality of life, microvascular complications, macrovascular complications, all-cause mortality, and cost (secondary outcomes). Randomized clinical trials (RCTs), quasi-RCTs, non-RCTs, controlled before-after, interrupted time series, cohort studies, and cost studies reporting data on these outcomes will be included. Eligibility will not be restricted by publication status, language of dissemination, duration of study follow-up, or time period of study conduct.

Two reviewers will screen the titles and abstracts resulting from the literature search, as well as potentially relevant full-text articles, in duplicate. Data will be abstracted and quality will be appraised by two team members independently. Conflicts at all levels of screening and abstraction will be resolved through team discussion.

Our results will be described narratively. Random effects meta-analysis and network meta-analysis will be conducted, if feasible and appropriate.

**Discussion:**

Our systematic review results can be used to determine the most effective, safe and cost-effective third-line strategies for managing T2DM. This information will be of great use to health policy-makers and clinicians, as well as patients living with T2DM and their families.

**Trial registration:**

PROSPERO registry number: CRD42013003624

## Background

Type 2 diabetes mellitus (T2DM) is a chronic condition characterized by insulin deficiency and impaired beta-cell function [1]. T2DM is the most common type of diabetes, accounting for 90% to 95% of all cases worldwide [2]. Over the past few decades, there has been a steady increase in the incidence and prevalence of T2DM, and it is estimated that 300 million individuals will have T2DM globally by 2025 [3]. These increased rates have been attributed to the aging population, obesity and a sedentary lifestyle [4].

First-line management of T2DM begins with lifestyle modifications, including diet and exercise, followed by the addition of pharmacological interventions in the form of oral hypoglycemic agents, namely metformin [5,6]. If these interventions are not successful, other oral hypoglycemic agents are usually initiated, with sulfonylureas being the medication of choice due to efficacy, availability and cost [6,7]. However, second-line management does not result in sustained diabetes control for all patients [8]. For these patients, third-line therapy with dipeptidyl peptidase-4 inhibitors (DPP-4 inhibitors; for example, sitagliptin, saxagliptin, linagliptin, vildagliptin) might offer hope.

DPP-4 inhibitors act by influencing glucagon-like peptide (GLP-1). GLP-1 is an incretin hormone released into circulation in response to nutrients. GLP-1 stimulates insulin secretion and decreases glucagon secretion [9]. It has a short half-life of one to two minutes and is rapidly broken down by DPP-4. DPP-4 inhibitors prevent the degradation of GLP-1 and thus, increase the action of incretin hormones [9]. However, DPP-4 is also responsible for the breakdown of other hormones and its inhibition has the potential to cause adverse events. As such, a key question posed by Canadian decision-makers pertains to which agents should be used for third-line therapy in patients whose glycosylated hemoglobin (A1C) level remains elevated. In particular, what are the comparative safety, effectiveness and cost-effectiveness of DPP-4 inhibitors compared with intermediate acting insulin for adults with T2DM?

## Methods/Design

A systematic review protocol was compiled, circulated for feedback from Canadian policy-makers, clinicians and systematic review methodologists, and revised as necessary. Subsequently, our protocol was registered with the PROSPERO database (CRD42013003624). Our systematic review plan conforms to the Preferred Reporting Items for Systematic reviews and Meta-analyses Protocols (or PRISMA-P) initiative [10]. Our methods are similar to a systematic review on type 1 diabetes mellitus that was submitted as a protocol publication to the *Systematic Reviews* journal (Tricco *et al*., personal communication), and will be described briefly here.

### Eligibility criteria

The eligibility criteria for our systematic review have been developed using the PICOS criteria [11], and are presented in Additional file 1.

#### Patients

Adults (≥18 years old) with T2DM who have used or are currently using metformin and another second-line agent (for example, sulfonylureas, glitazones, GLP-1 analogues, glinides, alpha-glucosidase) and have A1C ≥6.5%.

#### Interventions

DPP-4 inhibitors, including sitagliptin, vildagliptin, saxagliptin, and linagliptin.

#### Comparators

DPP-4 inhibitors compared with each other, intermediate-acting insulin (for example, neutral protamine Hagedorn (NPH), lente), no treatment, or placebo.

#### Outcomes

The primary outcome is A1C, while secondary outcomes include emergency department visits (ED), physician visits, hospital admissions, weight gain/loss, fractures, infections (for example, nasopharyngitis, pancreatitis), quality of life, microvascular complications (retinopathy, neuropathy, nephropathy), macrovascular complications (cardiovascular disease, stroke/transient ischemic attack, peripheral vascular disease), all-cause mortality, cost and cost-effectiveness.

#### Study designs

Experimental studies (randomized clinical trials (RCTs), quasi-RCTs, non-RCTs), quasi-experimentalstudies (controlled before after studies, interrupted time series), observational (cohort) studies and cost studies.

#### Other limitations

No other limitations will be imposed. As such, unpublished studies, studies written in all languages, studies of all durations and those conducted during all points in time are eligible for inclusion.

### Information sources and literature search

We will search a variety of sources to identify literature relevant to our PICOS criteria described above. First, we will search electronic databases (MEDLINE, EMBASE, and the Cochrane Central Register of Controlled Trials). Second, we will search for unpublished material via clinical trial registries (for example, World Health Organization International Clinical Trials Search Portal), public health agency websites (for example, Public Health Agency of Canada, Health Canada), drug regulatory organization websites (for example, Food and Drug Administration (FDA)), and conduct general searches in Google Scholar. Third, we will use other methods to ensure literature saturation, including searching the authors’ personal files, contacting DPP-4 inhibitor manufacturers, scanning the reference lists of included studies and relevant reviews [9,12], and contacting authors who publish frequently on T2DM and DPP-4 inhibitors.

All of the information sources will be searched by an experienced librarian affiliated with the Li Ka Shing Knowledge Institute of St. Michael’s Hospital. She has compiled a draft search for the MEDLINE database (OVID interface), which is presented in Additional file 1. This will be peer reviewed by another expert librarian affiliated with the Centre for Practice-Changing Research of the Ottawa Hospital Research Institute. She will use the Peer Review of Electronic Search Strategies (PRESS) checklist [13] for this process.

### Study selection process

We will pilot-test our screening criteria developed *a priori* among the team. We will develop a ‘cheat sheet’ with detailed definitions and examples to ensure high inter-rater reliability (that is, a kappa statistic ≥0.60 [14]) among the team. The eligibility criteria and cheat sheet will be revised, as necessary. After this calibration exercise, two team members will screen all of the literature search results in duplicate. Conflicts will be resolved by discussion among the team. The same process will be followed for potentially relevant full-text articles, as the screening criteria are slightly different for this level of screening [see Additional file 1]. All screening will be conducted using the online SysRev Tool [15], proprietary software developed by the Li Ka Shing Knowledge Institute of St. Michael’s Hospital.

### Data items and data collection process

To synthesize information on study characteristics, we will abstract data on the study design, country of conduct, year of conduct, DPP-4 inhibitor examined, and comparator(s) examined for each of the included studies. To summarize patient characteristics, we will abstract the type and number of patients, age (mean and standard deviation), baseline A1C, and co-morbidities for the included studies. Finally, we will abstract the results of the outcomes of interest, such as A1C, ED visits, physician visits, quality of life, adverse events, and cost at 6, 12 and 24 months, as well as the longest duration of follow-up.

To abstract the data, the team will use a rigorous process. A draft data abstraction form and ‘cheat sheet’ will be circulated among the team. Using a random sample of five to ten of the included studies, the data abstraction form will be pilot-tested by all team members. The form will be revised, as necessary, and data abstraction will begin when high inter-rater reliability (that is, a kappa statistic ≥0.60 [14]) has been achieved. Subsequently, two team members will abstract all of the included studies independently. Conflicts will be resolved by discussion.

We anticipate some challenges that might occur during the data abstraction process. For example, sometimes duplicate publications (or companion reports) that use data from the same group of patients are difficult to identify. To ensure the data is not double-counted in meta-analysis [16], the team will sort through the included studies to identify companion reports. We may also encounter poorly reported information and will have to contact authors for data clarifications when this occurs.

### Methodological quality/risk of bias appraisal

We will appraise the methodological quality or risk of bias of the included studies using tools that have been developed to assess the internal validity of different study designs. For example, the Newcastle-Ottawa scale was developed to appraise the methodological quality of observational studies, such as cohort studies [17]. The Cochrane Effective Practice and Organisation of Care Risk of Bias Tool was developed to assess the risk of bias of experimental and quasi-experimental studies [18]. We will also appraise publication bias using funnel plots [19] and studies reporting harms will be assessed using the McHarm tool [20]. All quality appraisal and risk of bias assessment will be conducted by two team members independently and conflicts will be resolved through team discussion.

### Synthesis of included studies

To synthesize our results, we will summarize all of the abstracted data. For example, we will provide overall summaries for each of the following: study characteristics, patient characteristics, methodological quality appraisal, and risk of bias assessment. We will observe the number of studies available for each of the outcomes examined, study designs by outcome, as well as the total number of patients included in the studies that report each outcome.

We will subsequently determine whether a random-effects meta-analysis [21] is feasible and appropriate. In order to assess this, we will examine the level of clinical, methodological, and statistical heterogeneity. The first two types of heterogeneity will be examined using the clinical and methodological insight of the research team. The third type of heterogeneity will be examined by looking for outliers in the forest plots, as well as by calculating an I^2^ statistic [22]. An I^2^ statistic ≥60% usually signifies moderate-to-high statistical heterogeneity [22].

If heterogeneity is identified and we have included at least 10 studies, we will conduct a meta-regression analysis to explain the observed heterogeneity [23]. Our meta-regression analysis will explore the influence of the following factors on our results: baseline A1C values (source of statistical heterogeneity); age, duration of diabetes (sources of clinical heterogeneity); and study quality (source of methodological heterogeneity). The frequentist meta-analysis and meta-regression analysis will be conducted in SAS Version 9.2 [24].

We anticipate that some of the included studies will not report measures of variance, including standard deviations, standard errors and 95% confidence intervals for continuous effect sizes. To make use of all existing data, these will be imputed using established methods [25]. The impact of these imputations will be calculated via a sensitivity analysis, which will allow the examination of missing data under both random and nonrandom assumptions [26].

After we have conducted the frequentist meta-analysis, we will attempt to conduct a network meta-analysis using WinBUGS [27]. For this analysis, median rankings (or effect sizes) and 95% credible intervals (interpreted the same way as 95% confidence intervals through frequentist methods) will be calculated [28]. A random effects network meta-analysis will be conducted, including all available direct and indirect data [28], as well as data from experimental, quasi-experimental, and observational (cohort) study designs. We will examine trace and history plots and calculate the Gelman Rubin statistic [29]. Consistency of results will be examined by comparing the frequentist meta-analysis results to those obtained through network meta-analysis, as well as using established statistical methods [30,31].

Finally, we will conduct numerous sensitivity analyses to test the robustness of our results. Examples include observing the influence of attrition, high risk of bias, and inclusion of different study designs (for example, cohort, quasi-experimental). We will also conduct a sensitivity analysis on the use of different priors for variance parameters included in the Bayesian network meta-analysis [28].

## Discussion

Our systematic review results have the potential to impact a large proportion of the population. The incidence of T2DM is increasing globally and it is estimated that 300 million individuals will have T2DM by 2025 [3]. Most patients with T2DM require second-line therapy [32], and some of these patients also require third-line treatment with DPP-4 inhibitors. Previous reviews of DPP-4 inhibitors have not specifically compared these agents with intermediate acting insulin [9,12]. This important topic was identified directly by health decision-makers in Canada.

Our systematic review results will be of great interest to key stakeholders, including the Canadian Drug Safety and Effectiveness Network, policy makers, researchers, clinicians (for example, College of Family Physicians) and patients. In addition to our integrated knowledge translation approach, a spectrum of end of grant knowledge translation strategies will be utilized. These will range from passive dissemination via publication in open-access journals and creation of knowledge tools, to more active activities, such as providing educational sessions. We will also hold an in-person dissemination meeting with key stakeholders, as well as create a one-page executive summary specifically targeting patients, policy-makers and clinicians.

## Abbreviations

A1C: glycosylated hemoglobin; DPP-4: dipeptidyl peptidase-4; ED: emergency department; FDA: Food and Drug Administration; GLP-1: glucagon-like peptide; PRESS: Peer review of electronic search strategies; PRISMA-P: Preferred reporting items for systematic reviews and meta-analyses protocols; RCT: randomized clinical trial; SysRev: systematic review; T2DM: type 2 diabetes mellitus.

## Competing interests

The authors declare that they have no competing interests.

## Authors’ contributions

ACT conceived the study, designed the study, helped obtain funding for the study and helped write the draft protocol. JA registered the protocol with the PROSPERO database and edited the draft protocol. CS edited the draft protocol. BH, DM, BH, CHY and SRM provided input into the design and draft of the protocol. SES conceived the study, designed the study, obtained the funding and helped write the draft protocol. All authors read and approved the final protocol.

## Supplementary Material

Additional file 1: Appendix Click here for file
