# Supplementary material for: Safety, effectiveness, and cost of dipeptidyl peptidase-4 inhibitors versus intermediate acting insulin for type 2 diabetes: protocol for a systematic review and network meta-analysis
Source: Syst Rev. 2013 Jun 28;2:47. doi: 10.1186/2046-4053-2-47 (PMC3702520; doi:10.1186/2046-4053-2-47)
Supplement: Additional file 1: Appendix [file 2046-4053-2-47-S1.docx]

**Appendix 1: Draft Eligibility Criteria**

Level 1 screening

1. Does this study include adult patients (aged ≥ 18 years) with type 2 diabetes?

YES____ NO____ UNCLEAR____

1. Is this an experimental, quasi-experimental, cohort or costing study?

YES____ NO____ UNCLEAR____

1. Are patients treated with DPP-4 inhibitors?

YES____ NO____ UNCLEAR____

| **Generic name** | **Trade name(s)** |
| --- | --- |
| Sitagliptin | Januvia |
| Vildagliptin | Zomelis, Galvus |
| Saxagliptin | Onglyza |
| Linagliptin | Tradjenta, Trajenta |

1. Does the study compare a DPP-4 inhibitor to another DPP-4 inhibitor, intermediate acting insulin preparations, or placebo?

YES____ NO____ UNCLEAR____

| **Generic name of agent** | **Trade name(s) of agent** |
| --- | --- |
| Isophane insulin (Neutral Protamine Hagedorn, NPH) | Novolin N, Humulin N |
| Zinc insulin (lente) | Humulin L, Novolin L |
| Sitagliptin | Januvia |
| Vildagliptin | Zomelis, Galvus |
| Saxagliptin | Onglyza |
| Linagliptin | Tradjenta, Trajenta |

- If you answer NO to any of these questions, the citation/study will be excluded. All other citations will be included.

Level 2 screening

1. Does this study include adult patients (aged ≥ 18 years) with type 2 diabetes who have A1C ≥7%?

YES____ NO____ UNCLEAR____

1. Is this an experimental, quasi-experimental, cohort, or costing study?

YES____ NO____ UNCLEAR____

1. Have patients been treated with metformin?

YES____ NO____ UNCLEAR____

1. Have patients been treated with sulfonylurea or other second-line agents (e.g., glitazones, GLP-1 analogues, glinides, or α-glucosidase)?

YES____ NO____ UNCLEAR____

1. Are patients being treated with DPP-4 inhibitors?

YES____ NO____ UNCLEAR____

| **Generic name** | **Trade name(s)** |
| --- | --- |
| Sitagliptin | Januvia |
| Vildagliptin | Zomelis, Galvus |
| Saxagliptin | Onglyza |
| Linagliptin | Tradjenta, Trajenta |

1. Does the study compare a DPP-4 inhibitor to another DPP-4 inhibitor, intermediate acting insulin preparations, no treatment, or placebo?

YES____ NO____ UNCLEAR____

| **Generic name of agent** | **Trade name(s) of agent** |
| --- | --- |
| Isophane insulin (Neutral Protamine Hagedorn, NPH) | Novolin N, Humulin N |
| Zinc insulin (lente) | Humulin L, Novolin L |
| Sitagliptin | Januvia |
| Vildagliptin | Zomelis, Galvus |
| Saxagliptin | Onglyza |
| Linagliptin | Tradjenta, Trajenta |

1. Does the study report at least one of the following outcomes?

Glycosylated hemoglobin (A1C); emergency department visits for hypo/hyperglycemia, physician visits for hypo/hyperglycemia, hospital admissions for hypo/hyperglycemia, weight gain, fractures, infections (e.g. nasopharyngitis, pancreatitis), quality of life, microvascular complications (retinopathy, neuropathy, nephropathy), macrovascular complications (cardiovascular disease, stroke/transient ischemic attack, peripheral vascular disease), all-cause mortality, cost or cost-effectiveness.

YES____ NO____ UNCLEAR____

- If you answer NO to any of these questions, the citation/study will be excluded. All other full-text articles will be included.

**Appendix 2: Draft MEDLINE literature search**

1 exp Diabetes Mellitus, Type 2/

2 exp Insulin Resistance/

3 T2DM.tw,ot.

4 NIDDM.tw,ot.

5 MODY.tw,ot.

6 "impaired glucose toleranc$".tw,ot.

7 (glucose adj intoleranc$).tw,ot.

8 (insulin$ adj resistanc$).tw,ot.

9 ("typ$ 2" adj2 diabet$).tw,ot.

10 ("typ$ II" adj2 diabet$).tw,ot.

11 ("typ$ 2" adj2 DM).tw,ot.

12 ("typ$ II" adj2 DM).tw,ot.

13 "non insulin$ depend$".tw,ot.

14 (noninsulin$ adj depend$).tw,ot.

15 noninsulin?depend$.tw,ot.

16 (non adj insulin?depend$).tw,ot.

17 ("keto$ resist$" adj diabet$).tw,ot.

18 (non?keto$ adj diabet$).tw,ot.

19 (adult$ adj2 diabet$).tw,ot.

20 (matur$ adj2 diabet$).tw,ot.

21 (late adj2 diabet$).tw,ot.

22 (slow adj diabet$).tw,ot.

23 (stabl$ adj diabet$).tw,ot.

24 ("insulin$ defic$" adj relative$).tw,ot.

25 (pluri?metabolic$ adj syndrome$).tw,ot.

26 or/1-25

27 Dipeptidyl-Peptidase IV Inhibitors/

28 gliptin?.mp.

29 sitaglipt?n$.mp.

30 saxagliptin.mp.

31 "BMS 477118".mp.

32 "BMS-477118".mp.

33 BMS477118.mp.

34 BMS-477118-11.mp.

35 UNII-9GB927LAJW.mp.

36 945667-22-1.rn.

37 onglyza.mp.

38 ondero.mp.

39 tradjenta.mp.

40 tra?enta.mp.

41 linagliptin.mp.

42 "BI 1356".mp.

43 BI-1356.mp.

44 "BI1356".mp.

45 668270-12-0.rn.

46 januvia.mp.

47 janumet.mp.

48 juvisync.mp.

49 ristaben.mp

50 tesavel.mp.

51 xelevia.mp.

52 (oral and "anti-hyperglyc?emic agent?").tw,ot.

53 sitagliptin.rn.

54 486460-32-6.rn.

55 "MK 0431".mp.

56 MK-0431.mp.

57 MK0431.mp.

58 HSDB 7516.mp.

59 UNII-QFP0P1DV7Z.mp.

60 vildaglipt?n.mp.

61 vildagliptin.rn.

62 vidagliptin.mp.

63 jalra.mp.

64 xiliarx.mp.

65 LAF237.mp.

66 LAF-237.mp.

67 UNII-I6B4B2U96P.mp.

68 galvus.mp.

69 NVP-LAF237.mp.

70 C502012.mp.

71 DPP-4.mp.

72 DPP4.mp.

73 DPPIV.mp.

74 DPP-IV.mp.

75 "dipeptidyl peptidase$ inhibitor$".mp.

76 "dipeptidyl-peptidase 4 inhibitor?".mp.

77 "dipeptidylpeptidase 4 inhibitor?".mp.

78 "dipeptidyl-peptidase IV inhibitor?".mp.

79 "dipeptidylpeptidase IV inhibitor?".mp.

80 or/27-79

81 exp Adult/ [ validated geriatric filter - best sensitivity ]

82 adult.mp. [ validated adult filter - best sensitivity ]

83 Middle Aged/

84 age$.tw.

85 or/81-84

86 26 and 80 and 85

87 exp Animals/ not (exp Animals/ and Humans/)

88 86 not 87
